# Predictive Modeling of Core–Shell Magnetoplasmonic Nanoparticles: GPR-Based Optimization for Enhanced Photothermal Heating

**DOI:** 10.1021/acsomega.5c09634

**Published:** 2025-12-13

**Authors:** Seda Aygul Akyuz, Zeliha Cansu Canbek Ozdil

**Affiliations:** Department of Materials Science and Nanotechnology Engineering, 52998 Yeditepe University, Istanbul 34755, Turkey

## Abstract

This study presents a comprehensive analysis of the photothermal therapy (PTT) performances of single gold (Au), single magnetite (Fe_3_O_4_), and Au@Fe_3_O_4_ and Fe_3_O_4_@Au core–shell nanoparticles using PyMieLab and COMSOL Multiphysics simulations. Through a detailed parametric study, we elucidate how core–shell configurations influence photothermal conversion efficiency, heat generation, and spatial temperature distribution. Our results reveal that Fe_3_O_4_ nanoparticles exhibit a higher intrinsic photothermal efficiency than Au; however, integrating both materials in core–shell structures markedly enhances thermal responses, with Fe_3_O_4_@Au consistently outperforming Au@Fe_3_O_4_. This enhancement arises from synergistic core–shell interactions that optimize light absorption and thermal conversion. A Gaussian process regression (GPR)-based efficiency parameter was introduced to predict optimal geometric combinations and temperature profiles. The analysis revealed clear design rules; the effective shell thicknesses ranged from 10 to 30 nm, while the most efficient core radius was around 80 nm. The highest predicted temperature elevations occurred at a 4:1 core-to-shell ratio, although the largest *J*_o_ values were observed at a ratio of 5:1. Among the optical parameters, the absorption cross-section (*C*_abs_) had the strongest influence on the photothermal performance, while *J*_o_, despite its limited predictive reliability in the current data set, remains a promising descriptor for future studies with broader material combinations. These findings underscore the pivotal role of nanoparticle design in PTT optimization.

## Graphical abstract

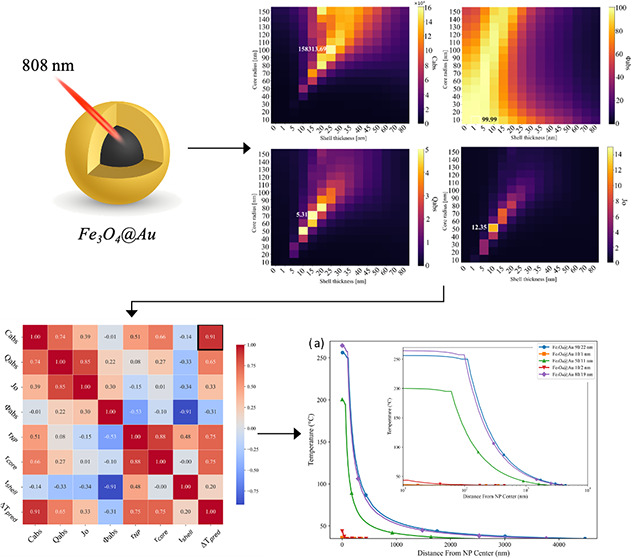

## 1 Introduction

The ability to customize optical properties is a key and distinctive feature of nanomaterials on the nanoscale. At this level, the interaction between the material and electromagnetic (EM) radiation greatly depends on the material’s type, size, and shape, as well as the properties of the medium through which the EM propagates.^1^ Metals that possess a negative real dielectric constant and positive imaginary dielectric constants can exhibit a special kind of interaction with EM radiation called surface plasmon resonance (SPR).^2^ SPR occurs due to the collective oscillation of surface electrons excited by EM radiation at the interface between the metal and the dielectric medium, resulting in a large absorption of EM radiation. The interaction of metal nanoparticles and EM waves is directly related to the optical properties of the material, which can be quantitatively expressed by the absorption cross-section (*C*_abs_), scattering cross-section (*C*_sca_), and extinction cross-section (*C*_ext_).^3^ The *C*_abs_ represents the absorption capacity of nanoparticles, which is particularly critical in PTT applications, as it enables the selective targeted heating of surrounding tissues to achieve the ultimate thermal destruction of host tumor cells or metastatic lesions. It is possible to precisely manage the converted thermal energy by varying the amount and duration of near-infrared (NIR) light exposure as well as the size and concentration of photothermal nanomaterials. For this purpose, various nanoparticles, from polymeric structures to metals such as gold and silver, are suitable for PTT applications.^4–9^ The *C*_sca_ indicates how efficiently nanoparticles can scatter light. In PTT, efficient scattering management is important for directing heat to the desired regions. The *C*_ext_ sum of the absorption and scattering cross sections evaluates the overall light-matter interactions of the nanoparticles. A high value indicates that the nanoparticles exposed to light exhibit strong absorption and scattering properties, resulting in an optical efficiency suitable for PTT applications.

The aforementioned interaction also generates a temperature increase in the nanoparticles, making them powerful nanoheaters.^4^ Thermoplasmonics is a branch of plasmonics that focuses on how much heat is generated and how to manipulate the nanoscale heat.^4,8^ Research in this field has become quite popular, notably in tumor therapy, drug delivery, sensing, solar energy harvesting, and surface-enhanced Raman scattering (SERS) applications.^4–7^

Especially, for PTT, the use of plasmonic nanoparticles is a notably effective therapy solution since the particles that accumulate on the tumor tissue only harm the nearby cancer cells when excited with a laser and leave the surrounding healthy tissues as undisturbed as possible.^8,9^ Cancerous cells have low heat tolerance; this technique shows notable promise. In PTT, which is considered a minimally invasive and specialized therapeutic capacity, the NIR laser is directed to the nanomaterials accumulated at the cancerous location.^8^ The NIR window, which is known as the phototherapeutic window, is used within the range of 650–1350 nm, where the laser has its maximum penetration depth and minimum interaction with the tissue.^10^

Anisotropic gold nanorods, bearing high aspect ratios, are valuable photothermal agents due to their adaptable optical properties in the NIR region (700–1100 nm), which is considered a PTT therapeutic window.^11–13^ However, these particles are difficult to produce in high yield and require toxic chemicals, such as cetyltrimethylammonium bromide (CTAB), during wet chemical synthesis to promote anisotropy.^13,14^ In contrast, isotropic nanomaterials overcome these challenges with simpler production methods and lower toxicity levels. Therefore, the search for biocompatible, isotropic PTT agents stands out as a more viable option in terms of both safety and environmental impacts.

For any nanoparticle to be used for in-body application, the particles should be traceable and create contrast in traditional imaging techniques such as magnetic resonance imaging (MRI) to ensure that the particles are successfully accumulated in the tumor region. Therefore, plasmonic nanoparticles can be coupled with magnetic nanoparticles to create magnetoplasmonic systems with advanced properties.^15–17^ Core–shell magnetoplasmonic systems based on gold and iron oxide nanoparticles are ideal theragnostic agents that can respond to both NIR light and magnetic fields, indicating their potential use in therapeutics and diagnostics.^18,19^

Table 1 provides a summary of the experimental studies focusing on the use of magnetic material-coated gold nanoparticles as PTT agents and vice versa. These studies highlight how the size of the core and shell influences the heat generation capacity of these systems, which is relevant for both photothermal and hyperthermia therapies. In these studies, the experiments focused on the heat generation efficiency of the materials, and a detailed size optimization process was not performed to obtain the highest generation capacity. Understanding the interactions between EM and nanoparticles is crucial for their intended applications, particularly those that use their optical properties. Theoretical calculation and numerical simulation techniques appear to be powerful and suitable techniques for achieving this goal. The Mie theory is widely used to investigate the interaction between spherical nanoparticles and EM waves.^3^ Previous studies, such as Chingsungnoen et al.,^24^ have examined Fe_3_O_4_@Au core–shell nanoparticles using the discrete dipole approximation (DDA) method, focusing on the tunability of optical absorption with respect to interparticle distance and shell geometry. They reported that coating spherical Fe_3_O_4_ nanoparticles with a thin Au layer in an aqueous medium induces a pronounced localized SPR response that can be tuned across a broad spectral range, extending from the visible to the NIR region.

**Table 1:** Summary of Au and Fe_3_O_4_-Based Magnetoplasmonic Nanoparticles as PTT Agents

NP type	NP size [nm]	laser properties (wavelength, power, exposure time)	Δ*T* (°C) (tissue/solution)	reference
Au@Fe_2_O_3_	20–50 nm	808 nm, 2 W/cm^2^; 5 min	∼12 ± 1.4 °C (tissue)	^20^
Fe_3_O_4_@Au	∼37 nm	808 nm, 1.4 W/cm^2^, 10 min	∼12 °C (tissue)	^21^
Au@MgFe_3_O_4_	42 ± 7 nm	808 nm, 1 W/cm^2^, 4 min	∼20 °C (tissue)	^22^
Fe3O4@Au	89 nm	980 nm, 1 W/cm^2^, 8 min	∼42.5 (solution)	^23^

In this study, we investigate the heat generation efficiency of magnetoplasmonic nanoparticles by using both PyMieLab software, which provides an analytical solution to the scattering and absorption of EM waves by spherical particles using Maxwell’s equations^3,15,25^ and the finite element method (COMSOL Multiphysics). Using PyMieLab, we compute key optical parameters, extinction, scattering, and absorption cross sections (*C*_ext_, *C*_sca_, *C*_abs_) as well as their corresponding efficiencies (*Q*_ext_, *C*_sca_, *Q*_abs_), absorption fraction (ϕ_abs_), and joule number (*J*_o_) based on Mie theory. We propose a new efficiency function derived from these optical features using a machine learning-based GPR model to identify key parameters governing photothermal performance and optimize nanoparticle architectures for maximum heat conversion by systematically varying core and shell dimensions. Subsequently, the thermal performance of the selected nanoparticle systems is further evaluated by using the Heat Transfer Module in COMSOL Multiphysics. The Heat Transfer in Solids (*ht*) and Radiative Beam in Absorbing Media (*rbam*) interfaces are coupled to simulate photothermal heating under optical excitation to observe temperature increase caused by both the SPR and the Beer–Lambert Law.

## 2 Computational Methods

Optical simulations are initially performed for homogeneous Au and Fe_3_O_4_ nanoparticles, followed by core–shell configurations of Au@Fe_3_O_4_ and Fe_3_O_4_@Au to calculate the *C*_abs_ and dimensionless efficiency parameters (*Q*_abs_, ϕ_abs_, and *J*_o_). These simulations aim to identify the optimal combinations of the core radius and shell thickness for enhancing the photothermal efficiency. For each configuration, the five highest and lowest values of the calculated efficiency parameters are compared under stationary heat simulation conditions, and a new efficiency calculation is generated with the GPR based on previously calculated efficiency parameters. Subsequently, time-dependent temperature analysis is realized, and temperature distributions over time profiles are extracted. The *C*_ext_, *C*_sca_, and *C*_abs_ are analyzed across a wide range of core radii from 10 to 150 nm with a step size of 10 nm and shell thicknesses from 0 to 80 nm with a step size of 1 nm.

### 2.1 Optical Simulations via PyMieLab

PyMieLab is an open-source simulation interface for homogeneous and core–shell spherical nanoparticle models based on the Mie-theoretic analysis of matter–light interactions as an EM beam of a given wavelength propagates along the *z*-axis, as shown in Figure 1. λ denotes the wavelength of the incident light, *r*_p_ is the radius of a homogeneous nanoparticle, whereas *r*_c_ represents the core radius of a core–shell nanoparticle, and *t*_s_ represents the shell thickness. The terms (*n*, *k*)_p_, (*n*, *k*)_c_, (*n*, *k*)_*s*_, and (*n*, *k*)_m_ refer to the complex refractive indices of the homogeneous particle, the core and shell refractive indexes of core–shell nanoparticles, and the surrounding medium, respectively.

**Figure 1:**
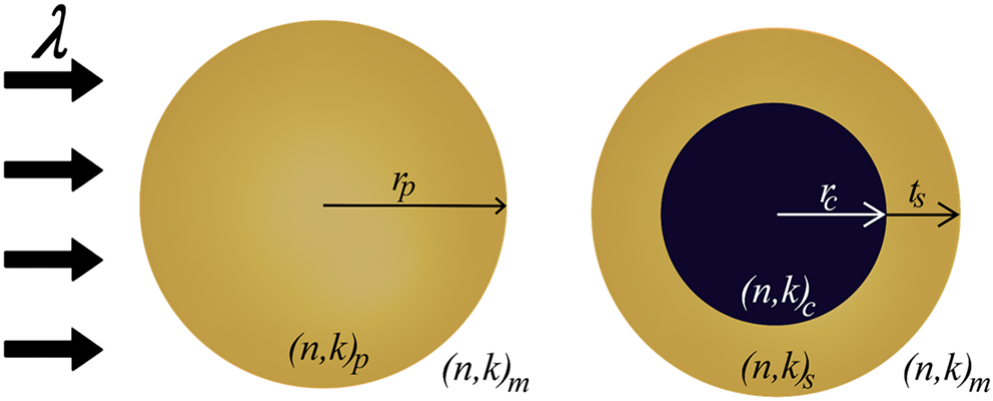
EM interaction of (left) homogeneous and (right) core–shell nanoparticle. Adapted with permission from ref 25.

The *C*_ext_, *C*_sca_, and *C*_abs_ values are calculated as follows^25^

1 $$C_{\mathrm{ext}}=\sum_{n=1}^{\infty }\left(2n+1\right)\left(a_{n}+b_{n}\right)$$

2 $$C_{\mathrm{sca}}=\frac{2\pi }{k^{2}}\sum_{n=1}^{\infty }(2n+1)\left({\left|a_{n}\right|}^{2}+{\left|b_{n}\right|}^{2}\right)$$

3 $$C_{\mathrm{abs}}=C_{\mathrm{ext}}-C_{\mathrm{sca}}$$

The scattering coefficients, *a*_*n*_ and *b*_*n*_, are calculated using Riccati-Bessel functions^25^

4 $$a_{n}=\frac{\mu m^{2}j_{n}\left(\mathrm{mx}\right){\left[x{\mathrm{J̇}}_{n}\left(x\right)\right]}^{\prime }-\mu_{1}j_{n}\left(x\right){\left[\mathrm{mx}{\mathrm{J̇}}_{n}\left(\mathrm{mx}\right)\right]}^{\prime }}{\mu m^{2}j_{n}\left(\mathrm{mx}\right){\left[xh_{n}^{\left(1\right)}\left(x\right)\right]}^{\prime }-\mu_{1}h_{n}^{\left(1\right)}\left(x\right){\left[\mathrm{mx}{\mathrm{J̇}}_{n}\left(\mathrm{mx}\right)\right]}^{\prime }}$$

5 $$b_{n}=\frac{\mu_{1}j_{n}\left(\mathrm{mx}\right){\left[x{\mathrm{J̇}}_{n}\left(x\right)\right]}^{\prime }-\mu j_{n}\left(x\right){\left[\mathrm{mx}{\mathrm{J̇}}_{n}\left(\mathrm{mx}\right)\right]}^{\prime }}{\mu_{1}j_{n}\left(\mathrm{mx}\right){\left[xh_{n}^{\left(1\right)}\left(x\right)\right]}^{\prime }-\mu h_{n}^{\left(1\right)}\left(x\right){\left[\mathrm{mx}{\mathrm{J̇}}_{n}\left(\mathrm{mx}\right)\right]}^{\prime }}$$

Here, *x* = 2π*rn*_*m*_/λ is defined as the size parameter. μ_1_ and μ represent the magnetic properties of the particle and medium, respectively. The functions *j*_*n*_(*mx*) and *h*_*n*_(1)­(*x*) represent the first and third species of the spherical Bessel function. These functions also serve as boundary conditions, because the values at the particle surface must be equal for the functions used inside and outside the particle. The implementation performs a simplification by assuming that μ_1_ and μ are equal to each other.

The absorption fraction, ϕ_abs_, is another metric used to evaluate the heat efficiency, which is calculated as follows^26^

6 $$\phi_{\mathrm{abs}}=\frac{C_{\mathrm{abs}}}{C_{\mathrm{ext}}}\times 100$$

Namely, ϕ_abs_ represents the percentage of extinguished light that is absorbed and converted into thermal energy, serving as a key indicator of the photothermal efficiency. A higher value indicates more efficient heat generation with minimal optical losses due to scattering.

The absorption efficiency *Q*_abs_ is calculated as follows

7 $$Q_{\mathrm{abs}}=\frac{C_{\mathrm{abs}}}{\pi r^{2}}$$

Here, *C*_abs_ is the previously calculated absorption cross-section area and π*r* ^2^ represents the particle’s geometrical cross-sectional area.^27,28^ In the field of nanoparticle optics, the *Q*_abs_ absorption efficiency defined in eq 7 is often used as a dimensionless indicator to assess how effectively a particle absorbs light, relative to its geometric cross-sectional area. While this metric provides insight into far-field absorption efficiency, it does not account for the volume of material involved or the near-field enhancement inside the nanoparticle, which is crucial for evaluating photothermal conversion. In contrast, the joule number, *J*_o_, addresses these limitations by incorporating both the particle’s absorption cross-section and the physical volume^28^

8 $$J_{o}=\frac{\lambda_{\mathrm{ref}}C_{\mathrm{abs}}}{2\pi V_{\mathrm{np}}}$$

where λ_ref_ is the reference wavelength (1242 nm) and *V*_*np*_ is the particle volume. This definition shifts the focus from surface interaction to volumetric heat generation efficiency, making it more appropriate for applications such as photothermal therapy, where the internal electric field and thermal response dominate. While both *Q*_abs_ and *J*_o_ are dimensionless and derived from the absorption cross-section based on nanoparticle dimensions, it is stated that *J*_o_ offers a material perspective, helping identify how efficiently the nanoparticle’s volume contributes to heat generation rather than quantifying how much light is absorbed relative to surface area.^28^

### 2.2 Heat Simulations via COMSOL Multiphysics

In heat simulations, a stationary study is initially realized, followed by a time-dependent study, allowing the system to evolve over a sufficiently extended period. The heat equation used in the COMSOL Heat Transfer in Solids *(ht)* interface is governed by eqs 9, 10 ^29^

9 $$\rho \;C_{p}\frac{\delta T}{\delta t}+\rho \;C_{p}u\cdot \Delta T+\nabla \cdot q=Q+Q_{\mathrm{ted}}$$

10 q=−k·ΔT

Here, ρ represents the material density and *C*_p_ represents the heat capacity at constant pressure. These values are taken from the COMSOL Material Library. The *u* denotes the velocity vector of the material in the medium, *T* the temperature, *q* the heat flux, and *k* the thermal conductivity. The initial temperature of the entire system is set to 308° K. This equation describes the heat transfer in the system, considering both time-dependent and spatial variations, particularly in the presence of a moving medium. The term $\rho C_{p}\frac{\delta T}{\delta t}$ represents the transient accumulation of thermal energy, accounting for the temperature changes over time. The second term ρ*C*_p_ *u*·Δ*T* accounts for convective heat transfer, where heat is transported by the movement of the fluid with velocity *u*, which is neglected. The ∇·*q* term corresponds to conductive heat transfer, which describes how heat diffuses through the material due to temperature gradients. On the right-hand side of the equation, *Q* denotes external or internal heat sources, such as laser energy input, while *Q*_ted_ accounts for additional heat generation mechanisms, such as thermoelastic damping or other coupled multiphysics effects. For these simulations, only the conductive heat transfer parameters, *Q* and *Q*_ted_, are taken into account.

Thermal insulator boundary conditions are applied to the outermost surfaces of the system to prevent heat transfer that is not included in the simulation environment. After obtaining the results, a *Surface-to-Ambient Radiation* boundary condition is applied to the outer boundaries of the simulation geometry. The Radiative Beam in Absorbing Media *(rbam)* interface is included in the model because the inspected nanoparticle size range is relatively large and the light absorption of particles through the Beer–Lambert effect might not be negligible at these sizes.

eq 11 describes how the laser beam intensity attenuates as it travels through an absorbing medium.^29^

11 $$\frac{e_{i}}{\mathrm{||}e\mathrm{||}}\cdot \nabla I_{i}=\kappa I_{i}$$

The term $\frac{e_{i}}{||e||}$ represents the unit vector in the direction of the laser beam propagation, ensuring that the intensity gradient is evaluated specifically along the beam path. When this unit vector’s dot product is taken with the gradient of the beam intensity, ∇*I*_*i*_, it extracts the directional rate of change of intensity along the beam’s trajectory. On the right-hand side of the equation, −κ*I*_*i*_, the exponential decay of beam intensity due to absorption is expressed, where κ is the absorption coefficient of the medium described as follows^29^

12 $$\kappa =\frac{4\pi k}{\lambda }$$

where *k* represented the imaginary part of the complex refractive index at the selected λ. In the case of gold, the refractive index is obtained from Johnson et al.,^30^ whereas for Fe_3_O_4_, the refractive index values are adopted from the work of Querry et al.^31^ In our simulations, the λ value is set to 800 nm. An additional heat source is incorporated using the *rbam* interface. Initial heat source is defined as a heat rate in the *ht* module caused by SPR by multiplying *C*_abs_ with an initial intensity value defined as 1 $\frac{\mathrm{mW}}{\mu m^{2}}$ .^32,33^

13 $$C_{\mathrm{abs}}\times I=P$$

Then, two *rbam* interfaces are defined separately for the core and shell domains. To account for the influence of the core on light absorption, two rbam interfaces are defined separately for the shell and core domains. For the shell, the incident light is applied along the *z*-axis with intensity *I*. For the core, the incident intensity is defined as the light transmitted from the shell to the core, calculated using Fresnel reflection and transmission principles via eqs 14 and 15.^34^ A secondary intensity is assigned to the shell to represent the portion of light reflected back from the core–shell interface (eq 15). This approach ensures that absorption in both the core and shell is accurately modeled, considering the bidirectional light transfer between the domains.^34^

14 $$trans\,\;shell\;to\;core=1-|\frac{\left(n_{shell}-n_{core}\right)}{\left(n_{shell}+n_{core}\right)}{|}^{2}$$

15 $$\mathrm{refl}\;\mathrm{core}\,\;\mathrm{to}\;\mathrm{shell}=|\frac{\left(n_{\mathrm{shel}l}-n_{\mathrm{core}}\right)}{\left(n_{\mathrm{shell}}+n_{\mathrm{core}}\right)}{|}^{2}$$

This approach enables a more accurate representation of EM interactions within the nanoparticle structure by accounting for multiple internal optical pathways.

In this study, the selected efficiency metrics, *C*_abs_, ϕ_abs_, *Q*_abs_, and *J*_o_, are compared by plotting heat maps using Python over different nanoparticle core/shell geometric combinations. To systematically explore the effect of these parameters, five extreme values representing the maximum and minimum limits are selected for each geometric parameter. The maximum temperature reached during the simulation is recorded for each configuration. The temperature elevation (Δ*T*) is calculated by subtracting the initial set temperature from this maximum value.

### 2.3 Machine Learning-Based Optimization of Core–Shell Nanoparticles

A Gaussian Process Regression (GPR) model is developed and trained to predict the stationary-state temperature rise of core–shell nanoparticles using optical and geometric parameters obtained from PyMieLab simulations. The output of the stationary heat transfer simulations is processed by subtracting the minimum temperature from the maximum temperature, and the resulting GPR-predicted temperature increase (Δ*T*_pred_) values are compiled together with the corresponding optical and geometric parameters. These parameters include *C*_abs_, ϕ_abs_, *Q*_abs_, *J*_o_, *r*_core_, *t*_shell_, and *r*_*NP*_ (total nanoparticle radius). Although *r*_core_ and *t*_shell_ determine *r*_*NP*_, all three are treated as separate input variables in our GPR-based model. This approach captures the effect of both individual and combined sizes on the optical response and mitigates overfitting, particularly in cases where different core–shell combinations yield identical total radii but distinct optical behaviors. These features form the input data set for the GPR model, while the simulated temperature change (Δ*T*_simulated_) values are used as the target variable.

An iterative sampling strategy is employed to enhance the model’s robustness and reduce uncertainty in critical regions. The model is initially trained on a base data set generated from various geometric combinations. After the first round of predictions, the geometric configurations yielding the largest prediction errors, identified by comparing Δ*T*_pred_ to the original simulation outputs, are examined in detail. To better represent these error-prone regions in the input space, the neighboring geometries of these outliers are added to the trained data set.

In the second iteration, new predictions are evaluated, and any significant discontinuities or gaps between Δ*T*_pred_ values are addressed by interpolating the intermediate geometries. These additional data points are simulated and appended to the data set, improving the model’s ability to generalize without bias toward sparsely sampled regions. Following this two-stage augmentation process, the final GPR model is trained on the enriched data set, resulting in a highly accurate, continuous, and smooth mapping from nanoparticle design parameters to thermal response.

The data set was split into training (80%) and testing (20%) subsets. A combination of statistical and visual methods is employed to validate the GPR model’s predictive performance. A 5-fold cross-validation approach is used to ensure generalization, where the model’s *R* ^2^ score and root-mean-square error (RMSE) are calculated for each fold. The feature response relationships are examined using LOWESS smoothing curves, and the parameter-based correlation matrix is analyzed to ensure interpretability and detect dominant influencing factors.

## 3 Results and Discussion

Figure 2 shows the variation of *C*_abs_, *C*_sca_, and *C*_ext_ for homogeneous Au and Fe_3_O_4_ nanoparticles with a radius of 10–150 nm within the wavelength range of 300–1000 nm. For both nanoparticles, the *C*_sca_ is clearly larger than the *C*_abs_. This suggests that most of the incident light will be scattered rather than absorbed. The *C*_abs_ for pure Au is highest at 480 nm for particles with a radius of 150 nm, whereas the Fe_3_O_4_ plasmon band is split into two modes. The highest absorption bands are observed at 349 and 569 nm for particles with a 150 nm radius. Neither of these materials exhibited an absorption profile in the NIR region. As a result, the effectiveness of both materials in photothermal therapy will be low due to the low *C*_abs_ and high *C*_sca_ in the NIR region.

**Figure 2:**
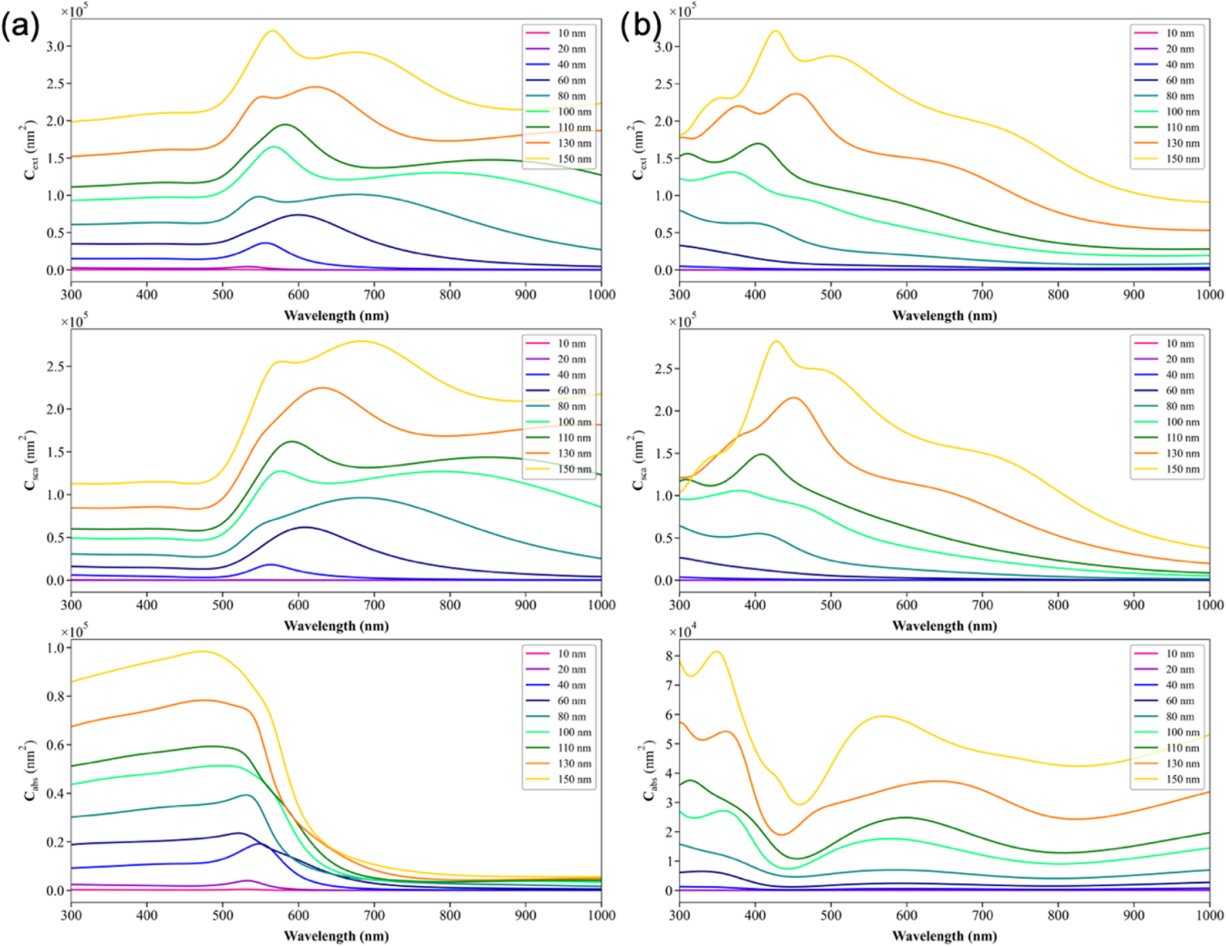
*C*_ext_, *C*_sca_, and *C*_abs_ values of (a) Au, (b) Fe_3_O_4_ of 10 to 150 nm radius nanoparticles.

Figure 3a shows the first derivative of the maximum optical intensities as a function of radius for Au nanoparticles and Figure 3b for Fe_3_O_4_ nanoparticles. For Au nanoparticles, both the extinction *C*_ext_ and scattering *C*_sca_ derivatives increase steadily with the particle size, indicating that larger Au particles exhibit significantly stronger scattering behavior. Notably, the growth rate of scattering consistently exceeds that of absorption, *C*_abs_, which highlights the dominant role of scattering in the optical response of Au nanoparticles as the size increases. In contrast, Fe_3_O_4_ nanoparticles display a different trend. At small particle sizes (below ∼20 nm), the derivative of absorption rises more sharply than that of scattering, indicating that Fe_3_O_4_ behaves predominantly as a material with a high absorptivity in this regime. Interestingly, the scattering derivative even begins with negative values, showing that increasing the radius initially leads to reduced scattering, unlike in Au. However, beyond approximately 20 nm, the scattering derivative surpasses the absorption derivative, reflecting a size-dependent transition in which scattering becomes more dominant.

**Figure 3:**
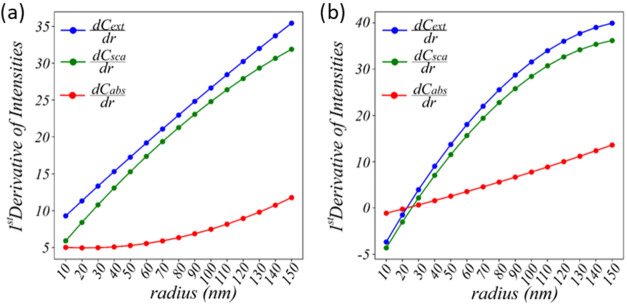
First derivative of the maximum intensities vs radius plot for (a) Au and (b) Fe_3_O_4_.

The variations of *C*_abs_, ϕ_abs_, *Q*_abs_, and *J*_o_, with respect to core radius and shell thickness for heterogeneous Au@Fe_3_O_4_ and Fe_3_O_4_@Au core–shell nanoparticles, are illustrated as matrices in Figures 4 and 5. For the Au@Fe_3_O_4_ configuration, the highest *C*_abs_ value of 122,291.5 nm^2^ was observed at a core/shell size of 110/80 nm. The maximum ϕ_abs_ of 99.62% occurred at 10/5 nm, while the highest *Q*_abs_ value of 1.10 was recorded at 100/80 nm. The maximum *J*_o_ value for this structure was 0.91, observed at 100/70 nm. In contrast, Fe_3_O_4_@Au nanoparticles exhibited a higher peak *C*_abs_ of 158,313.7 nm^2^ at 100/25 nm. The highest ϕ_abs_ was 99.99% at 10/1 nm. The maximum *Q*_abs_ value of 5.31 is found for particles with dimensions of 70/15 nm, and the highest *J*_o_ value of 12.35 is obtained at 50/10 nm.

**Figure 4:**
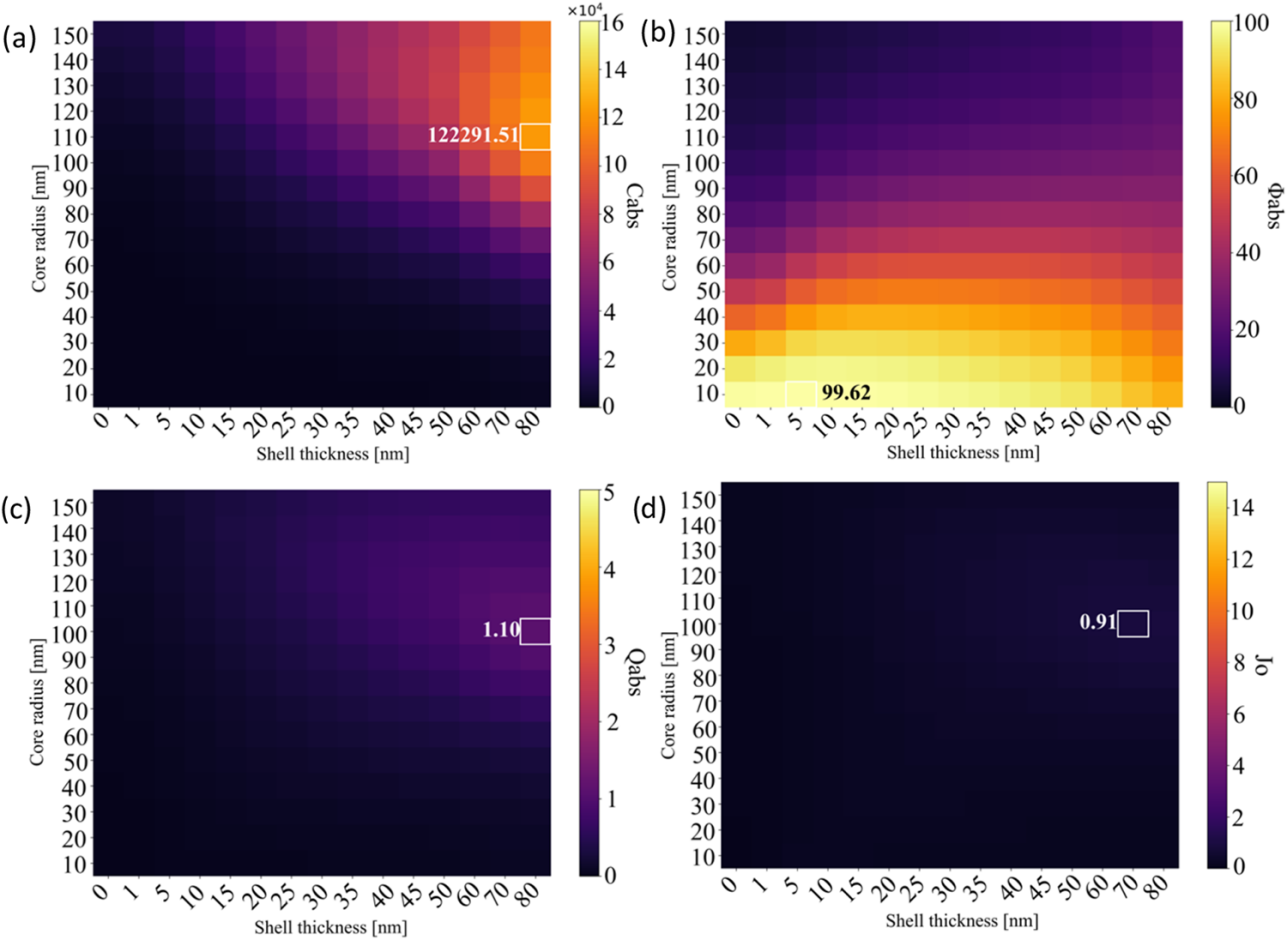
Variation of *C*_abs_, ϕ_abs_, *Q*_abs_, and *J*_o_ values at 800 nm wavelength for (a–d) spherical Au@Fe_3_O_4_.

**Figure 5:**
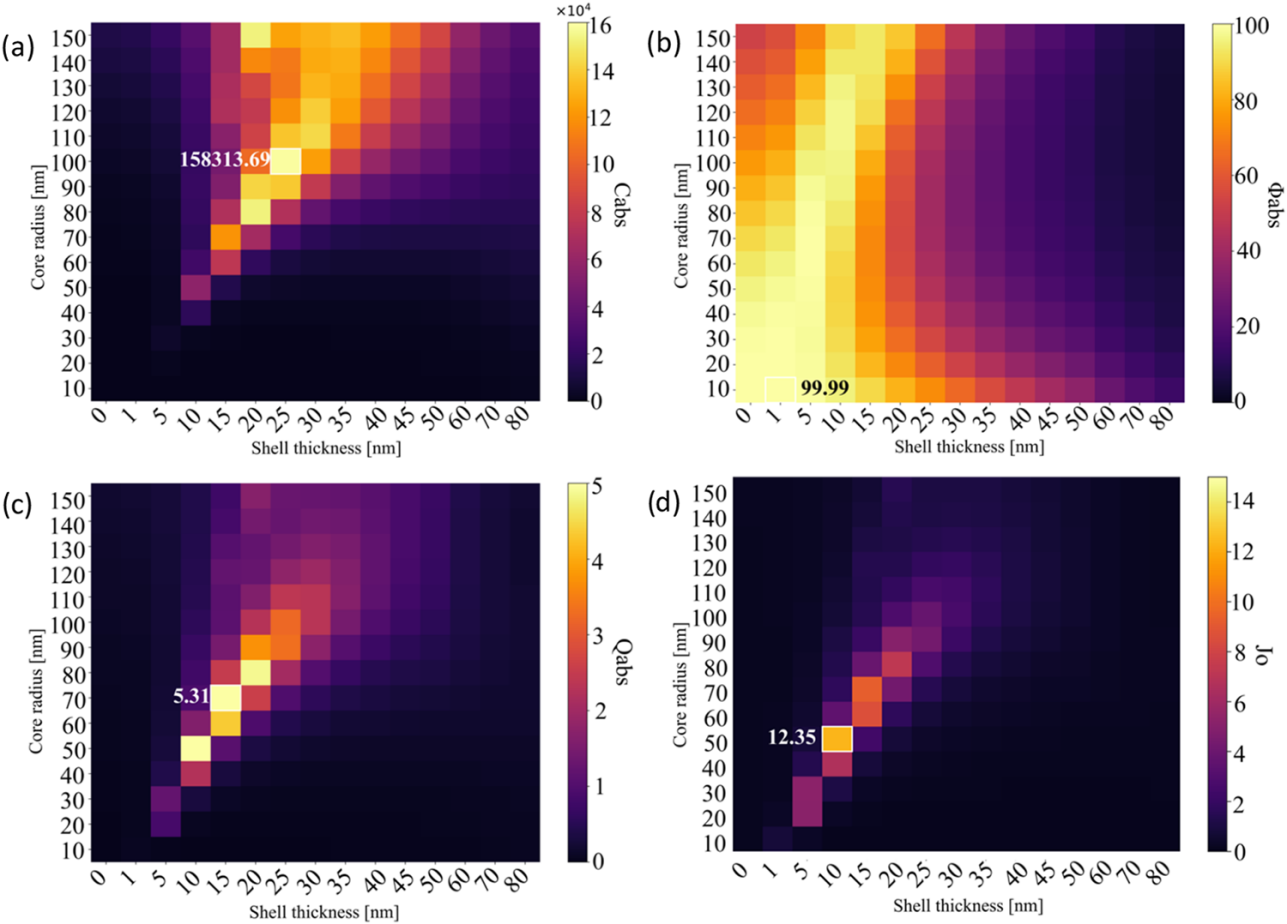
Variation of *C*_abs_, ϕ_abs_, *Q*_abs_, and *J*_o_ values at 800 nm wavelength for (a–d) spherical Fe_3_O_4_@Au.

Table 2 summarizes the highest obtained optical performance metrics for Au, Fe_3_O_4_, and various Fe_3_O_4_@Au and Au@Fe_3_O_4_ core–shell geometries and the calculated Δ*T*_simulated_ values. The obtained results show that Au nanoparticles have the lowest heat capacity due to their smaller *C*_abs_ values compared to Fe_3_O_4_ and core–shell nanoparticles. The 70/15 nm Fe_3_O_4_@Au system exhibits the highest absorption efficiency factor, indicating that it is a good absorber and a significant portion of the *C*_ext_ energy can be used for heat generation. With the highest *Q*_abs_ and *J*_o_ values, 100/25 nm, 80/20 nm, 70/15 nm, and 50/10 nm Fe_3_O_4_@Au nanoparticles are considered the best candidates for PTT. Au@Fe_3_O_4_ core–shell structures have lower *C*_abs_ and ϕ_abs_ values than Fe_3_O_4_@Au, resulting in lower heat generation capacity. The Fe_3_O_4_@Au nanoparticles exhibit higher Δ*T*_simulated_ values compared to those of Au@Fe_3_O_4_ and homogeneous counterparts. Accordingly, the training data set analyzed in the GPR model is constructed exclusively from Fe_3_O_4_@Au configurations, as this combination yielded the highest Δ*T*_simulated_ values in the simulations.

**Table 2:** Comparison of Heat Generation Efficiency Metrics of Homogeneous and Core–Shell Nanoparticles of Different Sizes for Static Conditions

structure	size (nm)	*C*_abs_ (nm^2^)	*C*_sca_ (nm^2^)	*C*_ext_ (nm^2^)	*Q*_abs_	ϕ_abs_ (%)	*J*_*o*_	Δ*T*_simulated_(°C)
Homogeneous								
Au	150	6.41 × 10^03^	2.27 × 10^05^	2.33 × 10^05^	0.09	2.75	0.56	5.30
Fe_3_O_4_	150	4.27 × 10^04^	1.05 × 10^05^	1.48 × 10^05^	0.60	28.89	3.75	36.46
Heterogeneous								
Au@ Fe_3_O_4_	110/80	1.22 × 10^05^	3.48 × 10^05^	4.70 × 10^05^	1.08	26.03	5.28	78.84
	100/80	1.12 × 10^05^	2.73 × 10^05^	3.85 × 10^05^	1.10	29.15	5.71	75.01
	100/70	9.51 × 10^04^	2.39 × 10^05^	3.34 × 10^05^	1.05	28.46	5.73	68.66
Fe_3_O_4_@Au	100/25	1.58 × 10^05^	2.17 × 10^05^	3.75 × 10^05^	3.23	42.16	24.01	153.05
	70/15	1.21 × 10^05^	4.54 × 10^04^	1.66 × 10^05^	5.31	72.65	58.14	171.58
	50/10	5.65 × 10^04^	7.00 × 10^03^	6.35 × 10^04^	5.00	88.97	77.48	114.55

Figure 6a presents the correlation matrix between each optical parameter and the corresponding Δ*T*_pred_. Among the evaluated parameters, *C*_abs_ exhibits the strongest correlation with Δ*T*_pred_. Figure 6b further illustrates this relationship, demonstrating that an increase in *C*_abs_ generally leads to a higher predicted Δ*T*_pred_. However, for identical *C*_abs_ values, variations in Δ*T*_pred_ are still observed across some data points. This indicates that, while *C*_abs_ is a dominant factor, other parameters, such as *r*_*NP*_, *r*_*c*ore_, and *Q*_abs_, also contribute significantly to the overall Δ*T*_pred_.

**Figure 6:**
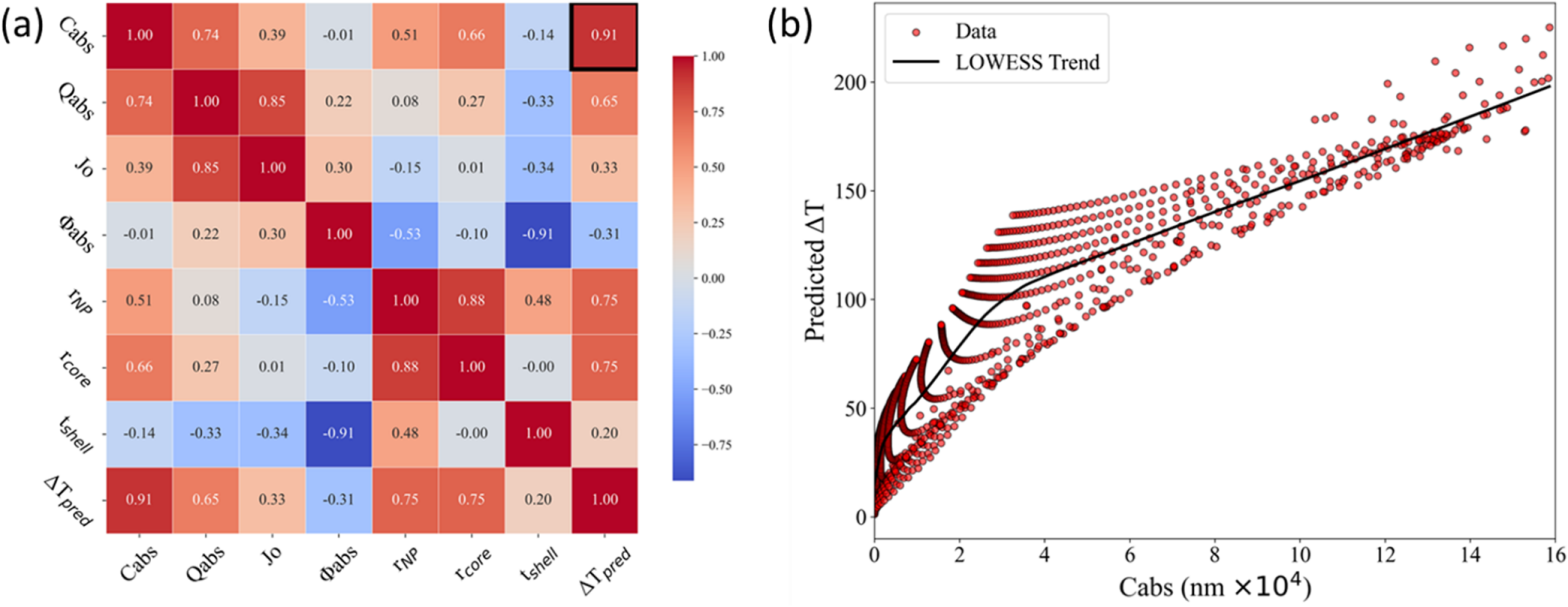
(a) Overall Correlation Matrix of the GPR Model. (b) Cabs vs predicted Δ*T* plotting for all PyMieLab Fe3O4@Au.

Table 3 further investigates this relationship by listing the geometric parameters *r*_*NP*_, *r*_core_, and *t*_shell_ alongside their corresponding optical responses and the resulting Δ*T*_pred_ values obtained from both GPR predictions and stationary heat transfer simulations. The analysis highlights that geometries optimized to maximize a single parameter, such as *C*_abs_, *Q*_abs_, and *J*_o_, do not necessarily yield the highest thermal performance. In other words, excelling in one optical descriptor alone is insufficient to achieve a maximum Δ*T*. This outcome is also illustrated in Figure 7, which plots wavelength versus cross-section values for each “maximum” case, showing that their thermal outputs remain suboptimal, while each geometry exhibits strong performance in its own metric. Furthermore, as shown in Table 3, parameters with weaker correlations to Δ*T* are associated with larger prediction errors, as reflected by the more pronounced differences between the predicted and the simulated values. Taken together, these findings confirm that relying solely on geometric tuning or the maximization of a single optical parameter cannot achieve optimal photothermal efficiency. Instead, the interplay between geometry and multiple optical factors must be considered simultaneously to design core–shell nanoparticles with superior Δ*T* values.

**Figure 7:**
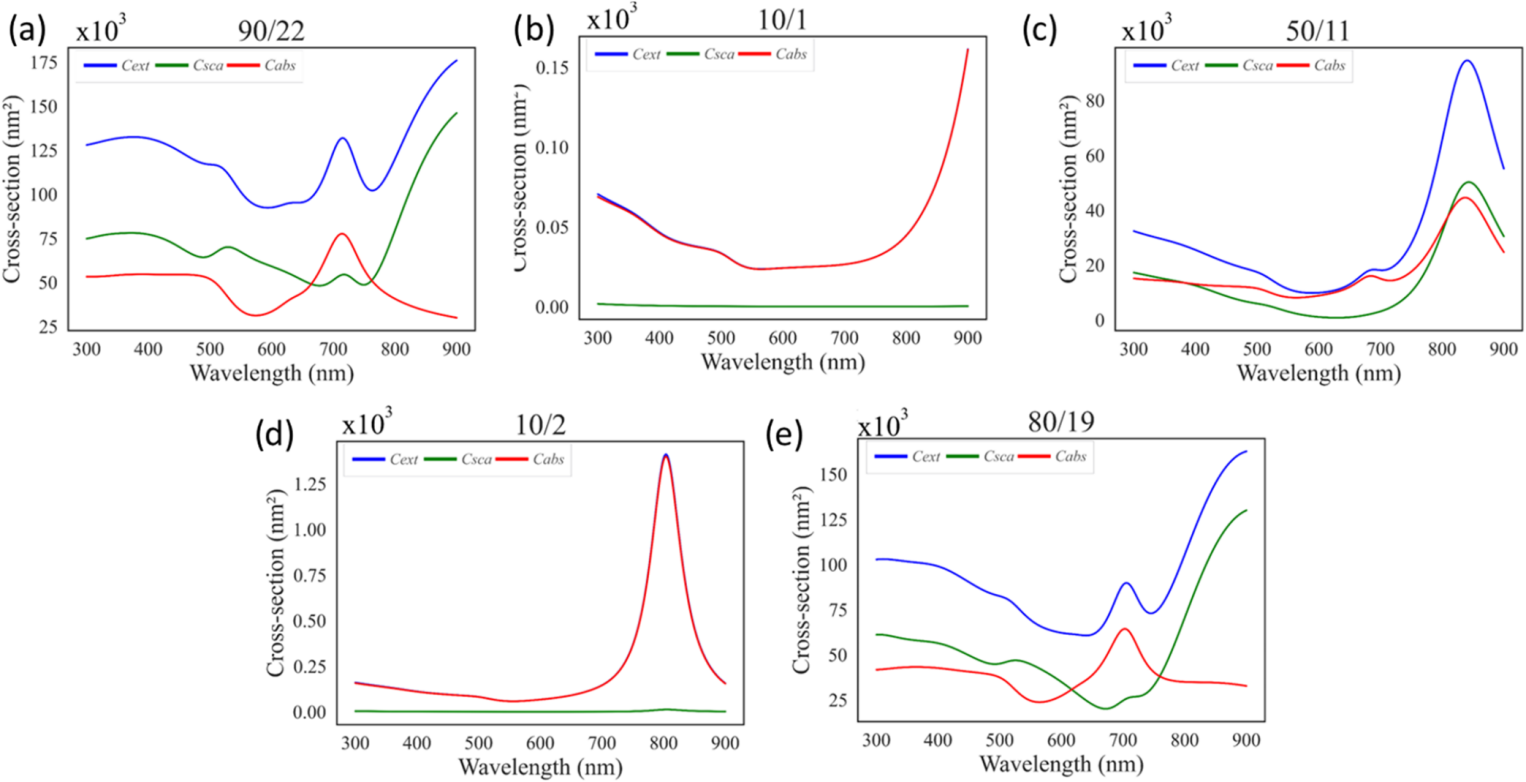
Maximum combinations of wavelengths vs cross-section values for (a) GPR predictions, (b) *C*_abs_, (c) *Q*_abs_, (d) *J*_o_, and (e) ϕ_abs_ Fe3O4@Au.

**Table 3:** Optical, Geometric, and Thermal Characteristics of the Nanoparticle Configurations Yield the Maximum Value for Each Efficiency Parameter and the GPR-Predicted Δ*T* Versus Simulated Δ*T*

structure	*r_NP_* (nm)	*r*_core_ (nm)	*r*_shell_ (nm)	*C*_abs_ (nm^2^)	ϕ_abs_ (%)	*Q*_abs_	*J*_o_	Δ*T*_pred_ (°C)	Δ*T*_simulated_ (°C)
*C*_abs_	112	90	22	1.63 × 10^05^	49.57	4.15	34.45	216.71	221.51
ϕ_abs_	11	10	1	2.23 × 10^01^	99.99	0.06	4.95	1.85	1.09
Q_abs_	61	50	11	7.42 × 10^04^	86.03	6.35	96.77	145.29	165.68
*J*_o_	12	10	2	7.07 × 10^02^	99.89	1.56	121.09	25.44	9.03
Δ*T*_pred_	99	80	19	1.59 × 10^05^	58.60	5.15	48.39	225.17	229.76

Further inspection of the parameter trends within the investigated range reveals clear optimal values for the Fe_3_O_4_@Au system. As shown in Figure 8, the most effective shell thickness values fall within the range 10–30 nm, while the most effective core radius lies between 50 and 100 nm, with 80 nm identified as the optimal value. The distribution of core sizes around this peak is not symmetrical; as the core radius increases beyond 80 nm, the absorption also increases due to the Beer–Lambert effect, which explains the observed differences compared with smaller core radii. Table 4 further confirms that the highest Δ*T*_pred_ values consistently occur when the core-to-shell dimensional ratio is close to 4:1. This ratio corresponds to the shell thickness that maximizes the predicted thermal response for each core radius evaluated. Moreover, as the ratio deviates further from 4:1, the discrepancy between Δ*T*_pred_ and Δ*T*_simulation_ increases, indicating reduced predictive accuracy and less efficient thermal performance.

**Figure 8:**
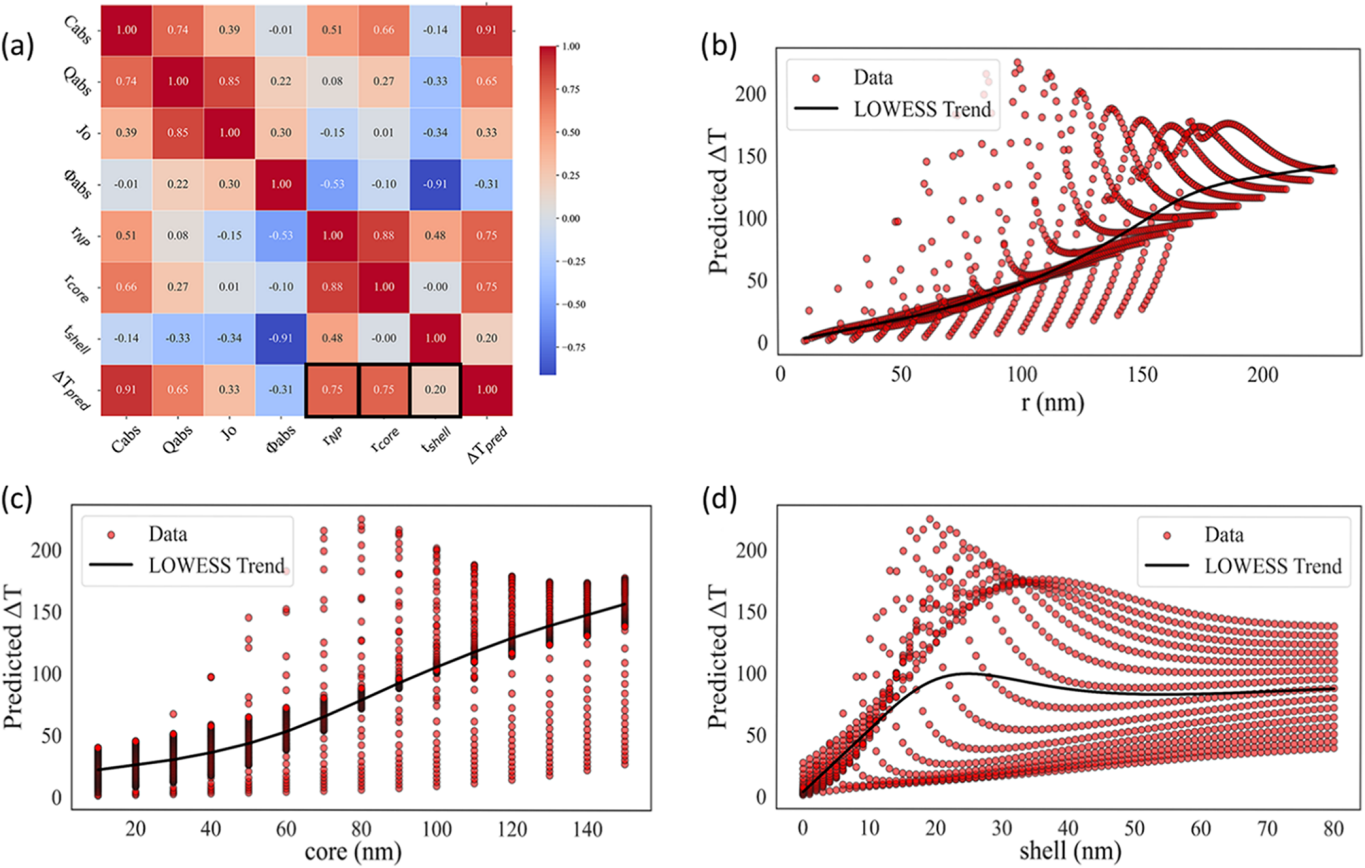
Δ*T* distribution with respect to (a) shell thickness, (b) core radius, and (c) nanoparticle radius. (d) Maximum Δ*T* value combinations for core radius 50–110 nm.

**Table 4:** Optimum Ratios of Core–Shell Dimensions for Various Nanoparticle Dimensions and Their Corresponding Δ*T* Prediction and Simulation Values

*r*_NP_ (nm)	*r*_core_ (nm)	*r*_shell_ (nm)	*r*_core_ (nm)/ *r*_shell_ (nm)	Δ*T*_pred_ (°C)	Δ*T*_simulated_ (°C)
138	110	28	3.93	188.26	188.51
125	100	25	4.00	201.77	203.37
112	90	22	4.09	216.71	221.51
99	80	19	4.21	225.17	229.76
86	70	16	4.38	215.81	226.27
73	60	13	4.62	183.26	199.13
61	50	11	4.55	145.29	165.68

When further discussing the features used in the training model, the ϕ_abs_ term is particularly noteworthy. As shown in both Tables 2 and 3, ϕ_abs_ can be misleading. Although ϕ_abs_ values appear numerically high, the actual *C*_abs_ values are negligible, particularly when evaluated at their peak wavelengths, as illustrated in Figure 9. This indicates that a high efficiency index, ϕ_abs_, does not necessarily correspond to a meaningful absorption peak. In these cases, both scattering and absorption cross-sectional values are extremely small, leading to inflated efficiency ratios.

**Figure 9:**
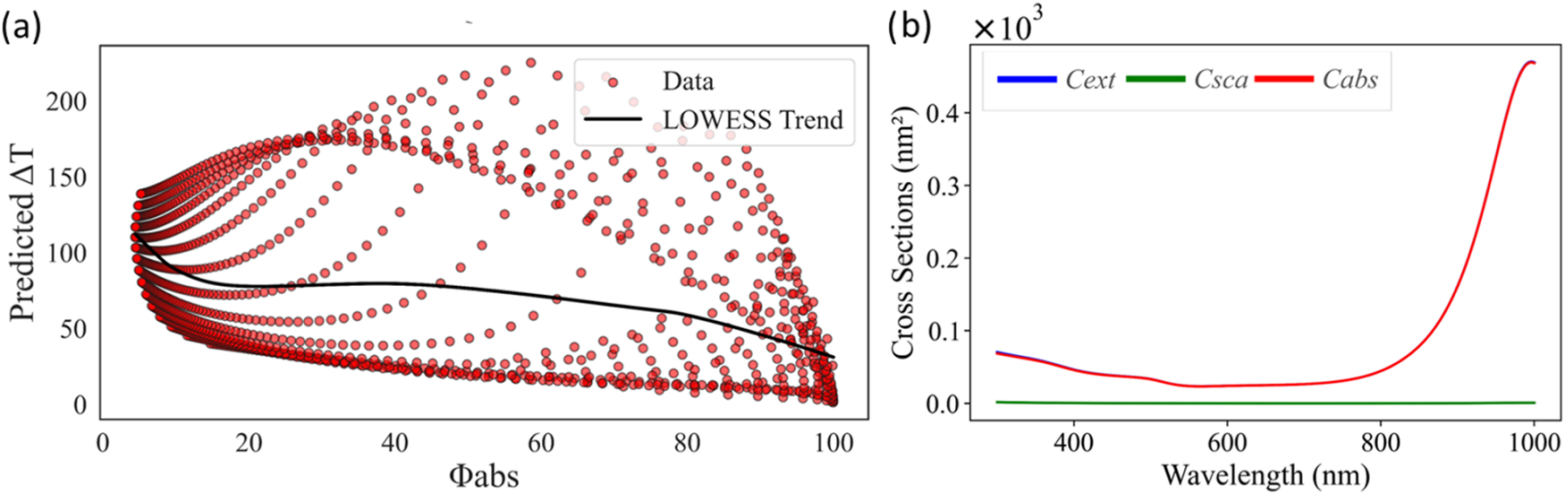
(a) Δ*T* distribution with respect to ϕ_abs_. (b) Cross-section values from PyMieLab for the maximum ϕ_abs_ combination.

When the correlation between Δ*T*_pred_ and *Q*_abs_ is examined, the distribution of data points across varying *Q*_abs_ values is shown in Figure 10a. In this data set, most points are concentrated at low *Q*_abs_ values, making it difficult to assess the effectiveness of this parameter; its influence could change if higher *Q*_abs_ cases are included. Within the inspected range, however, such conditions are not achieved for the Fe_3_O_4_@Au system. This observation is further supported by the standard deviation of the maximum values, which deviates from the fitted LOWESS trend curve. Unlike ϕ_abs_, the *Q*_abs_ parameter reflects a balance between *C*_abs_ and *C*_sca_, as illustrated in Figure 10, and therefore represents a more reliable efficiency factor than absorption alone. Interestingly, the top three *Q*_abs_ values correspond to some of the smallest particle dimensions listed in Table 4, indicating that even relatively small nanoparticles can generate a promising temperature increase in the system.

**Figure 10:**
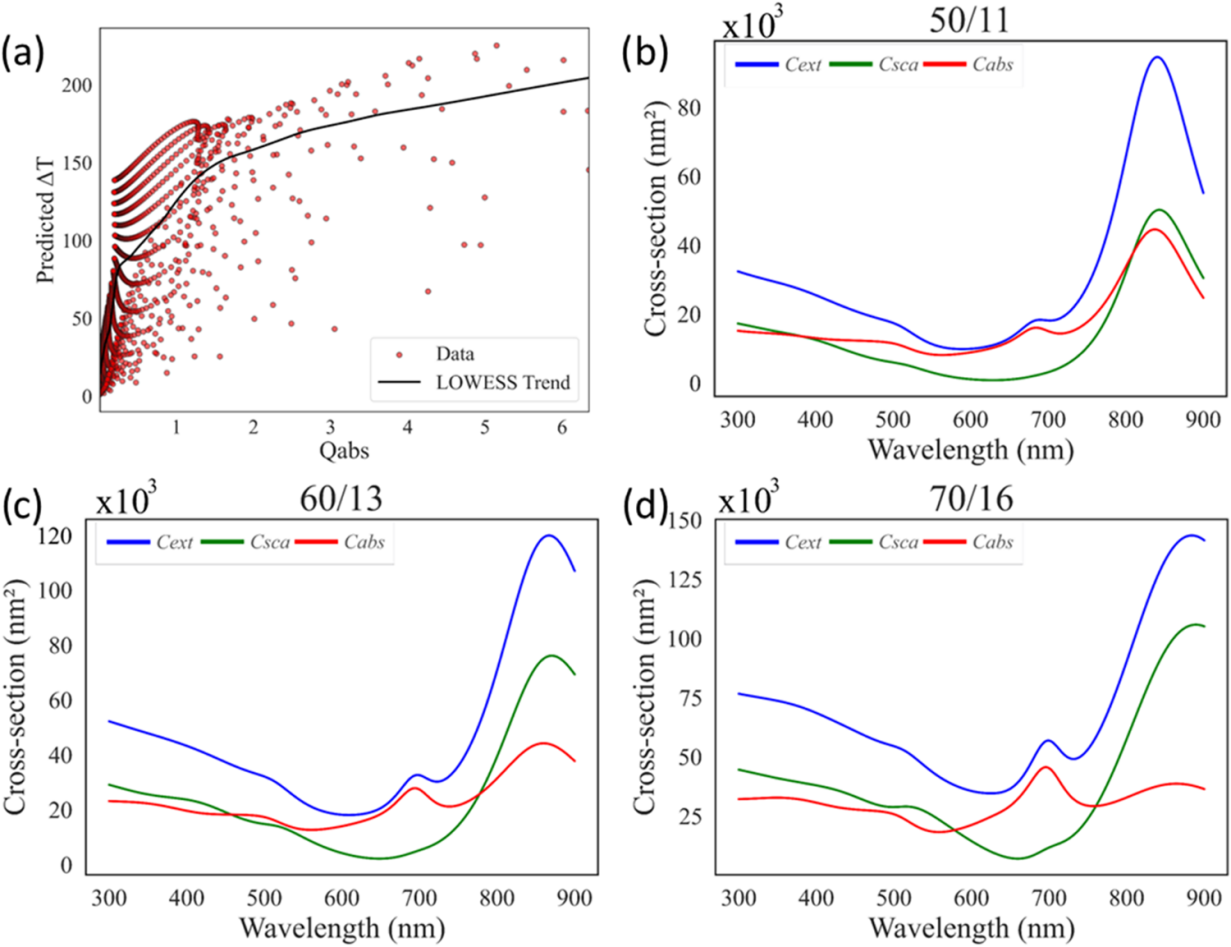
(a) Δ*T* distribution with respect to *Q*_abs_. Cross-section values from PyMieLab for the maximum *Q*_abs_ combination for (b) the maximum *Q*_abs_ value at 50/11, (c) the 2nd maximum *Q*_abs_ value at 60/13, and (d) the maximum 3rd *Q*_abs_ value at 70/16.

Another parameter evaluated for efficiency was *J*_o_, with its Δ*T*_pred_ distribution shown in Figure 11a. When the wavelength versus cross-section plots of the highest-performing combinations are examined, *J*_o_ emerges as the most effective indicator for identifying configurations with maximum absorption and minimum scattering. However, its direct influence on Δ*T*_pred_ is difficult to model reliably within the current data set. This limitation stems from two factors: (i) the majority of data points are concentrated at lower *J*_o_ values, and (ii) the higher *J*_o_ values exhibit the largest standard deviations among all investigated parameters. This effect arises because, with increasing particle diameter, the scattering rate grows significantly faster than the absorption rate, as discussed before. Conversely, tiny particles cannot produce the desired temperature increase under the specified light intensity values.

**Figure 11:**
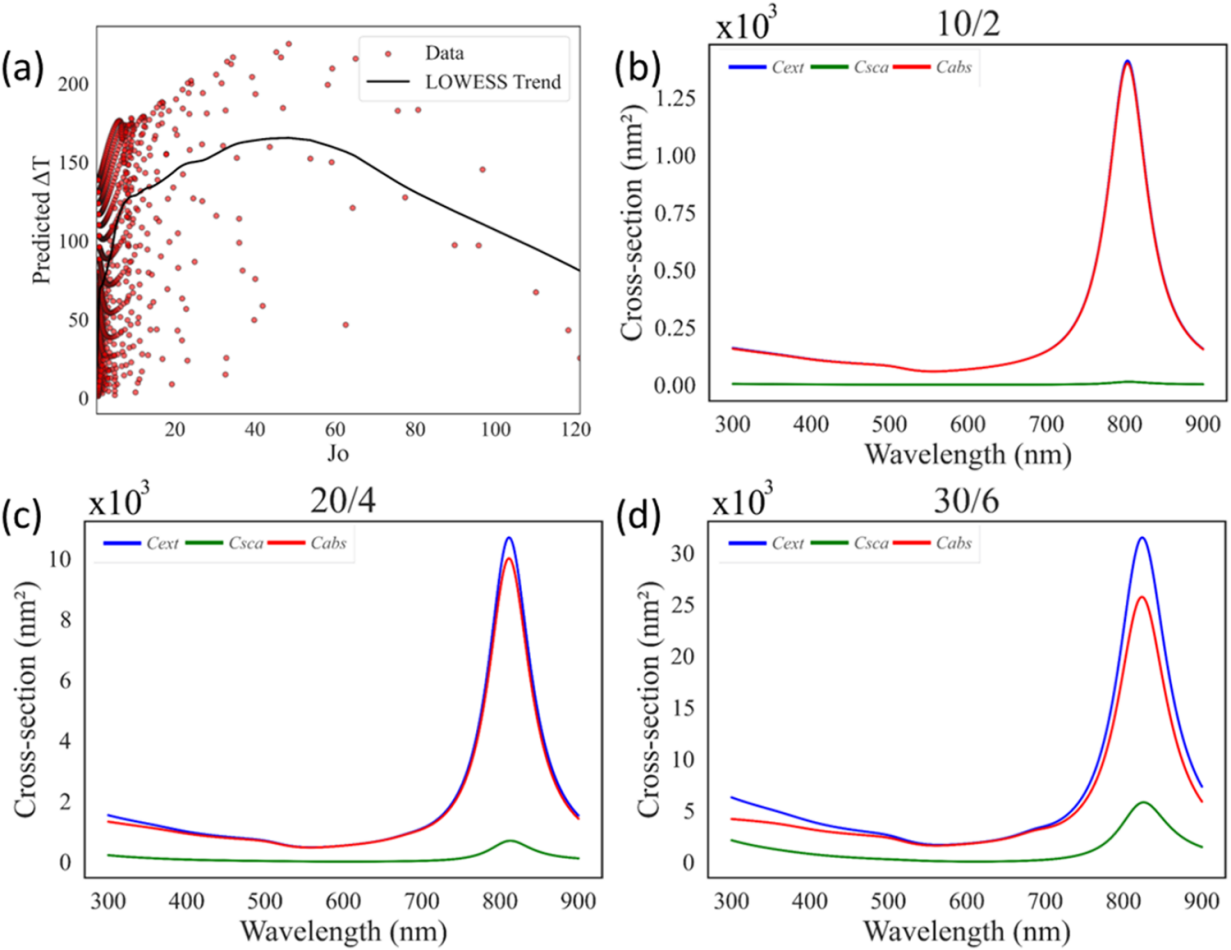
(a) Δ*T* distribution with respect to *J*_*o*_. Cross-section values from PyMieLab for the maximum *J*_*o*_ combination for (b) the maximum value at 10/2, (c) the 2nd maximum value at 20/4, and (d) the 3rd maximum value at 30/6.

Although the 80/20 geometric combination is not the best option for *C*_abs_, ϕ_abs_, *Q*_abs_, or *J*_o_, it performs best according to the newly proposed metric. This distribution suggests that while the Fe_3_O_4_@Au material combination offers certain benefits, it does not produce consistently high absorption at 800 nm for larger core–shell geometries. Consequently, the predictive power of *J*_o_ in relation to Δ*T*_pred_ remains underutilized in this study, and the training model is more focused on *C*_abs_, *r*_*NP*_, *r*_core_, and *Q*_abs_ parameters. Future analyses incorporating broader data sets, especially those featuring diverse material combinations, are needed to explore the full potential of *J*_o_, ideally, yielding a more uniform spread of *J*_o_ values that correlate more robustly with temperature increases. Also, in the wavelength versus cross-section plotting, peak values that have higher *C*_abs_ values at 700 nm were observed, which suggests that Fe_3_O_4_@Au combinations are more suitable for 700 nm laser applications rather than 800 nm.

After examining the influence of individual features on Δ*T*, the model’s predictive behavior is further evaluated using residual plots for two different training strategies, as shown in Figure 12. In Figure 12a, the model is trained only on extreme geometric cases, leading to overfitting. The LOWESS trend (red dashed line) shows systematic deviations with underestimation at low Δ*T*_pred_ and overestimation at high Δ*T*_pred_. The data set is iteratively expanded by adding intermediate geometries in regions of high prediction error, which significantly reduces bias (Figure 12b). The residuals in Figure 12b are more randomly distributed around zero, and the LOWESS trend remains nearly flat, indicating that the overfitting observed in Figure 12a is mitigated and the model is generalized better across the Δ*T*_pred_ range.

**Figure 12:**
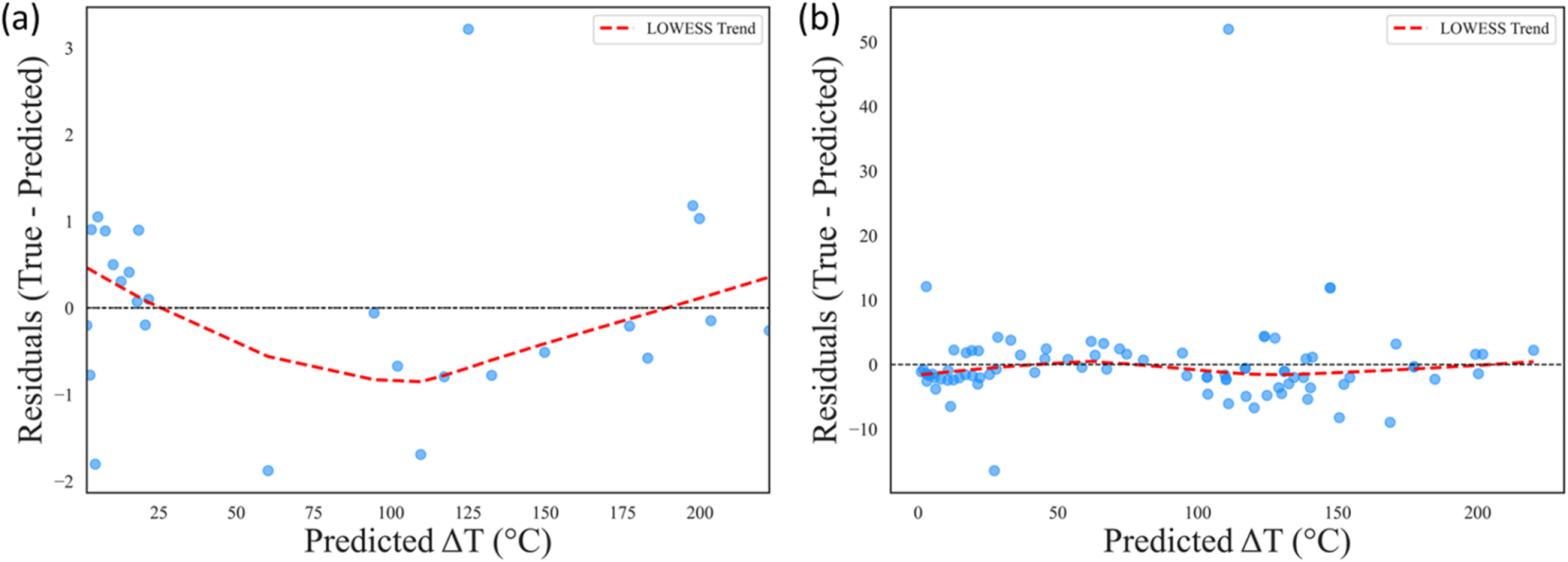
(a) Residual plots for Δ*T*_pred_: (a) overfitting when trained on extreme cases only. (b) Reduced bias and improved generalization after iterative data set expansion.

Figure 13 shows the local temperature distributions obtained using COMSOL Multiphysics Heat Transfer Simulation at an 800 nm wavelength for water at the distance from the nanoparticle center. In all systems, the highest temperature increase is observed near the nanoparticle center with an exponential decline in the surrounding medium. Figure 13a shows that the high-temperature region is limited to the immediate vicinity of the nanoparticle, minimizing thermal effects on the surrounding medium. The plot is presented as a subplot with the *x*-axis transformed into a logarithmic scale to aid interpretation.

**Figure 13:**
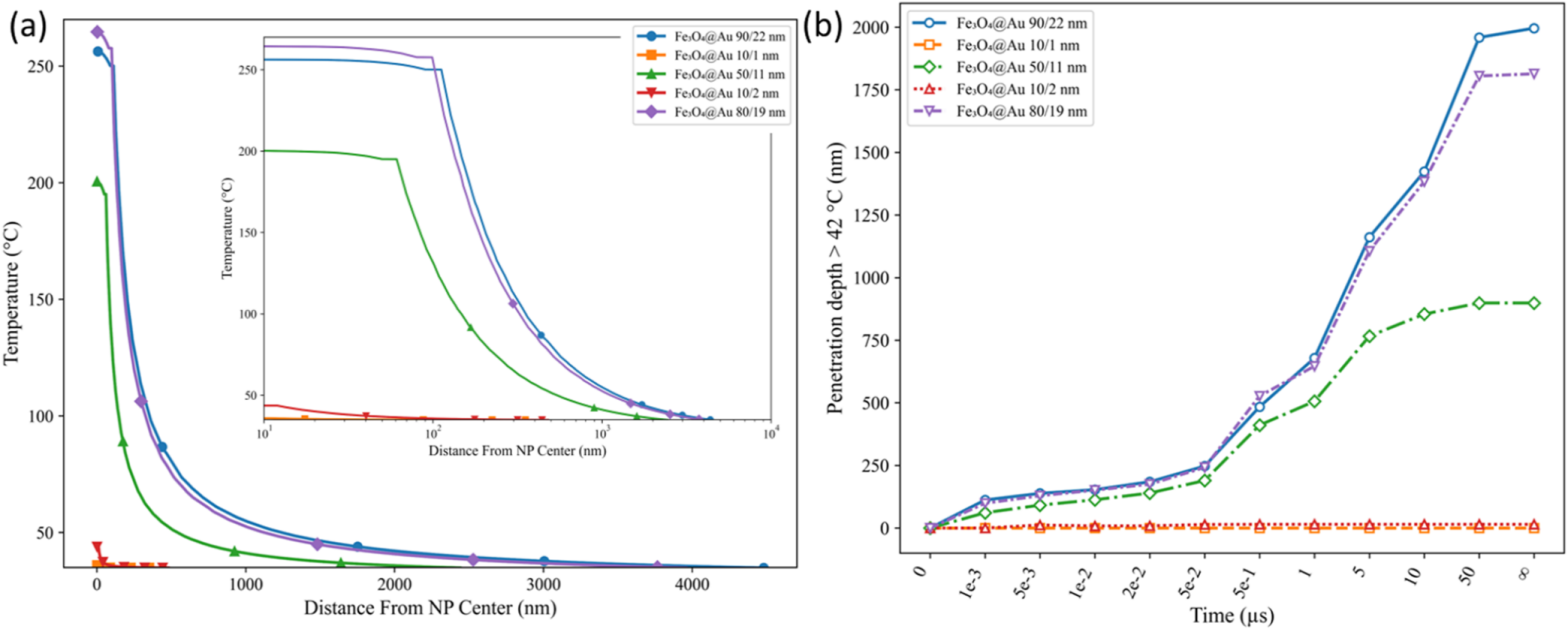
(a) Temperature profiles from the nanoparticle center to surroundings for stationary heat transfer in solid-radiative beam in absorbing media coupling, at optimal efficiency parameters (*C*_abs_, ϕ_abs_, *Q*_abs_, and *J*_o_) and GPR prediction. (b) Thermal penetration depth from the nanoparticle center in a water environment for heat transfer in solid-radiative beam in absorbing media coupling, showing regions where temperatures exceed 42 °C (hyperthermia threshold).

Among all of the selected particles, the highest temperatures were achieved with the 80/19 nm nanoparticles, which is the best value for the GPR prediction model. The best value for *J*_o_ remained much smaller than the other candidates because of its small particle size. It can also be seen that smaller particles decay faster. For further inspection, the time-dependent simulation results are plotted in Figure 13b. The 90/22 nm configuration exhibits the greatest penetration depth due to its larger dimensions, producing hyperthermia over a region with a diameter of approximately 3.99 μm. In comparison, the 80/19 nm configuration results in a slightly smaller affected area with a diameter of about 3.63 μm. Furthermore, the 50/11 nm configuration reaches its saturation point faster than others, suggesting that smaller particle volume is also important for PTT since too small particles might reach their saturation points before exceeding hyperthermia ranges, as can also be seen for 10/2 nm.

## 4 Conclusion

In conclusion, this study provides a comprehensive analysis of the PTT performances of single Au, single Fe_3_O_4_, Au@ Fe_3_O_4_, and Fe_3_O_4_@Au core–shell nanoparticles by using PyMieLab and COMSOL Multiphysics simulations. This parametric study elucidates the impact of core/shell configurations on the photothermal conversion efficiency and heat generation performance of the nanoparticles. Additionally, we propose a novel efficiency parameter derived from a GPR model, enabling the prediction of optimal geometric combinations and corresponding temperature profiles. Our findings demonstrate that the core–shell configuration significantly impacts the thermal response, with Fe_3_O_4_@Au core–shell nanoparticles exhibiting a superior heat generation efficiency. Fe_3_O_4_ nanoparticles showed higher photothermal efficiency than Au nanoparticles when compared to the heat generation of single-component nanoparticles.

However, integrating Fe_3_O_4_ and Au in core–shell configurations led to markedly enhanced thermal responses, with Fe_3_O_4_@Au outperforming Au@Fe_3_O_4_. This enhancement is attributed to the synergistic interaction between the core and shell materials, which optimizes light absorption and thermal conversion. Although the correlation heatmap initially suggested that core and shell radii were not individually dominant factors, a more detailed analysis revealed clear patterns. Optimal shell thicknesses were consistently found to be between 10 and 30 nm, and the most effective core radius peaked at 80 nm. The highest predicted temperature increase occurred at a core-to-shell ratio of approximately 4:1 across all inspected geometries.

Among the evaluated optical parameters, absorption cross-section *C*_abs_ had the most significant influence on the photothermal performance. However, in this study, the *J*_o_ parameters could not be effectively utilized in the model training, primarily because the data set included only a limited range of high *J*_o_ values. Moreover, *J*_o_ exhibited the highest standard deviation among the parameters, further complicating its predictive reliability in this specific data set. Despite these limitations, *J*_o_ remains a promising parameter, as it serves as a strong indicator for identifying geometric configurations that minimize scattering while maximizing absorption. Its full potential in predicting photothermal performance may become more apparent in future studies involving expanded data sets with diverse core–shell material combinations. Interestingly, although the optimal temperature elevation occurred at a 4:1 core-to-shell ratio, the highest *J*_*o*_ values were observed at a 5:1 ratio.

The COMSOL Multiphysics simulations provided insights into the spatial temperature distributions around the nanoparticles, further confirming the superior performance of core–shell structures with the highest thermal output and slower heat dissipation in both environments. The localized heating effect, critical for effective PTT, is most pronounced in Fe_3_O_4_@Au nanoparticles, making them highly suitable for targeted cancer therapy applications. Time-dependent simulations revealed that the nanoparticles reached their peak temperature within the microsecond range. Notably, the most efficient configuration, an 80 nm core with a 19 nm shell thickness, demonstrated the potential to induce hyperthermia in particles with diameters up to around 4 μm. Furthermore, our results emphasize that in systems with substantially larger core radii, photothermal modeling should account not only for SPR heating but also for volumetric absorption effects. To address this, we incorporated the Radiative Beam in Absorbing Media interface within the Heat Transfer in Solids module, enabling a more comprehensive representation of both surface and volumetric energy deposition.

These findings underscore the importance of nanoparticle design in optimizing the PTT efficiency. The core/shell architecture of Fe_3_O_4_@Au nanoparticles offers a promising approach to enhancing photothermal conversion and achieving effective hyperthermia treatment. Additionally, this predictive framework establishes quantitative design rules for magnetoplasmonic nanoparticle optimization toward PTT, beyond the purely optical characterization presented in earlier works.

Future studies should focus on experimental validation and exploration of surface modifications and functionalization strategies to further improve the biocompatibility and targeting capabilities of these nanoparticles for clinical applications and simulations of different material combinations.

## Nomenclature

*C*_abs_: absorption cross-section
*C*_sca_: scattering cross-section
*C*_ext_: extinction cross-section
ϕ_abs_: absorption fraction
*J*_o_: joule number
*a*_*n*_, *b*_*n*_: scattering coefficients
μ_1_: magnetic property of particle
μ: magnetic property of medium
*Q*_abs_: absorption efficiency
*V*_np_: particle volume
ρ: material density
*c*_p_: heat capacity
*u*: velocity vector of the material in the medium
T: temperature
*q*: heat flux
*k*: thermal conductivity
κ: absorption coefficient
*r*_core_: core radius
*t*_shell_: shell thickness
*r*_NP_: total nanoparticle radius
Δ*T*_pred_: GPR-predicted temperature increase
Δ*T*_simulated_: simulated temperature increase
